# Determination of standard molar volume of 1-hexyl-3-methylimidazolium bis(trifluoromethylsulfonyl)imide on titanium dioxide surface

**DOI:** 10.3389/fchem.2024.1416294

**Published:** 2024-06-20

**Authors:** Nannan Ye, Zhengxing Dai, Yifeng Chen, Xiaoyan Ji, Xiaohua Lu

**Affiliations:** ^1^ State Key Laboratory of Materials-Oriented Chemical Engineering, College of Chemical Engineering, Nanjing Tech University, Nanjing, China; ^2^ CAF, Key and Open Lab Forest Chem Engn, Key Lab Biomass Energy and Mat, Institute Chem Ind Forest Prod, SFA, Natl Engn Lab Biomas, Nanjing, China; ^3^ Division of Energy Science/Energy Engineering, Lulea University of Technology, Lulea, Sweden; ^4^ Suzhou Laboratory, Suzhou, China

**Keywords:** standard molar volume, colligative properties of solution, archimedes drainage method, Gay-Lussac pycnometer, [Hmim][NTf_2_], p25

## Abstract

The fluids near the solid substrate display different properties compared to the bulk fluids owing to the asymmetric interaction between the fluid and substrate; however, to the best of our knowledge, no work has been conducted to determine the interfacial properties of fluids experimentally. In this work, we combined a pycnometer with experimental measurements and data processing to determine the standard thermodynamic properties of interfacial fluids for the first time. In the study, 1-hexyl-3-methylimidazolium bis(trifluoromethylsulfonyl)imide ([Hmim][NTf_2_]) and titanium dioxide (P25) were chosen as the probes to prove the concept. It was found that, with the combination of the Gay-Lussac pycnometer and the colligative law, together with selecting a suitable solvent, it is possible and reliable to determine the standard molar volume of the immobilized [Hmim][NTf_2_]. Compared to the bulk phase, the molar volumes of [Hmim][NTf_2_] on the P25 surface reduce by 20.8%–23.7% at temperatures from 293.15 to 323.15 K, and the reduction degrees decrease with increasing temperatures. The newly determined standard thermodynamic data was used to obtain the model parameters of hybrid electrolyte perturbed-chain statistical associating fluid theory density functional theory (ePC-SAFT-DFT), and further predictions of the density of interfacial ionic liquids with different film thicknesses were proved to be reliable in comparison with the experiment results.

## 1 Introduction

With the development of novel nanomaterials, involving the interface (fluids with solids) to intensify the process performance has become a feature of modern industries (Amoo et al., 2022; Cole and Syres, 2022). The introduction of interface leads to significant changes in the properties of the fluids due to strong asymmetric and unbalanced molecular interactions (Migliorati et al., 2022). It has been demonstrated that interface or confinement typically results in significant alterations to the state, structure, and properties of ionic liquids (ILs), which are markedly distinct from those of bulk IL (Yoshida et al., 2019), including thermodynamic and kinetic properties (Malali and Foroutan, 2017; Zhang et al., 2021). For instance, the thermopower of ILs confined in ZIF-8 is considerably greater than that of bulk ILs; the ion transfer properties of ILs confined in MOF differ from those of bulk ILs due to the increased mobility of anions and cations under confinement (Zhang Z. J. et al., 2022; Qian et al., 2023; Liu et al., 2024); the anions and cations of the ILs on the surface of titanium dioxide have different orientations and arrangements from those of the bulk ILs (Dai et al., 2018). Molecular calculations indicate that the ILs confined in IRMOF−1 exhibit an ordered structure (Chen et al., 2011). Furthermore, density functional theory combined with experimental characterization reveals that the ILs confined in Cu-BTC form two different types of ion-pair structures due to interactions (Dhumal et al., 2016). There are notable variations in the wettability and adhesion between the ILs and titanium dioxide surfaces with varying degrees of roughness (An et al., 2013). However, no experimental methods have been proposed and developed to quantify the thermodynamic properties of the fluids on the surface (i.e., interfacial fluids). It is thus necessary to explore experimental methods to determine the thermodynamic properties of interfacial fluids (Wu et al., 2017a; Dong et al., 2020).

The methods and theories for obtaining the standard thermodynamic properties of bulk fluids have been very well established. Taking the Henry’s constant as an example (Kazarina et al., 2023), the infinitely dilute state is set as the reference and standard states, and the Henry’s constant is obtained from the experimental data by taking the limit of zero composition (Ivanov, 2018) based on the law of colligative properties of solutions (Aghaie et al., 2019; Chen et al., 2021). Principally, such a method can be used to determine the thermodynamic properties of the interfacial fluids, for example, the ILs immobilized on the solid surface. On the one hand, in the experiment, density (volume) is an easily measurable thermodynamic property. Anton Paar densitometer is a suitable high-precision measuring device, and its measurement relies on the oscillation of the U-shaped borosilicate glass tube in an undamped harmonic fashion under the excitation of electrons when filled with liquids (Mills and Vandevoort, 1981; Francesconi and Comelli, 1994). However, for a heterogeneous system, such as the system containing immobilized fluids, it often leads to accumulating and depositing over time, which makes it impossible to use the Anton Paar densitometer to determine the density of the immobilized fluids. On the other hand, the pycnometer has a high degree of accuracy in determining the density of solids. Among them, the Gay-Lussac pycnometer, prepared from high borosilicate glass and equipped with a recessed glass stopper of approx. 1 mm capillary diameter and a flat top, possesses an expanded uncertainty of 2 × 10^−4^ g·cm^−3^ in density (Kabo et al., 2004; Lorefice et al., 2014). Pycnometer has been used to determine the density of solids and liquids. Also, the average density of the water inside the pores was determined through pycnometer based on the assumption that the properties of H_2_O were affected only in the pores but not in the interfacial regions between particles (ETZLER & FAGUNDUS, 1983; Etzler and Fagundus, 1987). However, to determine the interfacial properties of fluids, it remains a challenge to select a suitable solvent for determining the volume of the sample without affecting the state of the fluids at the interface.

Among the studied fluids, ILs have been proposed as one of the most promising, advanced, and designable liquid materials, owing to their unique properties (Editorial, 2019; Siami et al., 2023), and received great attention (Wang et al., 2019; Liu et al., 2020). Incorporating ILs on the surface of substrates to create an interfacial effect and then achieve desirable performance has been widely proposed when developing advanced technologies for different applications, including batteries for energy storage, high voltage supercapacitors, and catalysis (Zhang Q. D. et al., 2022; Bahaa et al., 2024; Mousavi et al., 2024). To promote the development of such technologies, it is essential to obtain and understand the properties of ILs on a solid surface, which is also beneficial for developing thermodynamic models. The IL 1-hexyl-3-methylimidazolium bis(trifluoromethylsulfonyl)imide ([Hmim][NTf_2_]) is commercially available with stable properties, low viscosity, as well as easy preparation and purification features, and based on experiments, simulations, and characterizations, their properties, such as electrical conductivity (Papović et al., 2017a), density, viscosity (Papović et al., 2016), heat capacity, melting point (Papović et al., 2017b), refractive index, surface tension, self-diffusion coefficient, and sound velocity, have been systematically studied (Aljasmi et al., 2022).

Here, for the first time, an innovative method was developed to determine the standard molar volume (density) of immobilized ILs based on the measurements of the systems with different IL-film thicknesses and the definition of the reference and standard states as the IL-film thickness tending to zero. In the experiment, the classical Archimedes drainage method was combined with an inventive design of insoluble immobilized IL-solvent systems to accurately determine the density. In the theory, the principle of colligative properties was used to conduct data processing and obtain the standard molar volume via taking the limit of zero thickness. To illustrate the developed method, titanium dioxide (P25) nanoparticle was used as the substrate, [Hmim][NTf_2_] was chosen as the fluids, and squalane that is stable and insoluble with [Hmim][NTf_2_] was selected as the solvent. To validate the accuracy of the experimental data, standard thermodynamic data was used to obtain the parameters of the ePC-SAFT-DFT model developed in our group to describe the inhomogeneous properties of fluids on the surface of substrates. The model was further used to predict the density of the interfacial ILs at different film thicknesses, and the experimental results and model predictions were compared.

## 2 Materials and methods

### 2.1 Chemicals

The chemicals used in this work are summarized in Table 1. [Hmim][NTf_2_] was stored in a desiccator with color-changing water-absorbing silica gel. Prior to the experiment, the IL was treated in a vacuum drying oven at 343.2 K for 48 h to remove the residual water, and then the water content of [Hmim][NTf_2_] was measured by the TitroLine^®^ 7500 KF trace moisture analyzer, revealing a water content of 37 ppm. P25 (mass fraction purity ≥99.5%) was purchased from Degussa, and it was dried and degassed under vacuum at 353.2 K for 10 h before use. The ultrapure water (conductivity = 0.056 μS·cm^−1^ at 293.2 K) was prepared in the laboratory using the EPED laboratory-grade ultrapure water machine (PLUS-E2-10TJ). After preparation, the ultrapure water was heated to boiling for 10 min, ultrasonically processed to remove bubbles, and cooled for later use.

**TABLE 1:** Summary of the used chemicals.

Chemical name	Supplier	CAS No.	Mass fraction (purity)
[Hmim][NTf_2_]	Lanzhou Institute of Chemical Physics, Chinese Academy of Sciences	382,150–50–7	≥99%
Squalane	Acros Organics	111–01–3	≥99%
Ultrapure water	-	7,732–18–5	—
P25	Degussa	13,463–67–7	≥99.5%
Potassium bromide	Sigma-Aldrich	7,758–02–3	Spectral grade
Methanol	Lingfeng Chemical Reagent Co., China	67–56–1	≥99.7%

### 2.2 Preparation of P25-[Hmim][NTf_2_]

The IL was immobilized on the surface of the substrate (P25 in this work), and its amount was precisely determined. Briefly, [Hmim][NTf_2_] was immobilized into P25 particles according to the impregnation–vaporization method (Xie et al., 2016; Liu et al., 2018). Firstly, [Hmim][NTf_2_] was dissolved into the anhydrous methanol at room temperature stirring for 30 min, and then P25 particles were added into the solutions with different mass ratios and stirred for 2 h. After vacuum evaporation, the samples were dried at 353.2 K in a vacuum oven for 10 h. Depending on the IL-loading, the prepared samples were labeled as P25-[Hmim][NTf_2_]-*x*, where *x* represents the mass fraction of the IL, and stored in the laboratory desiccator. The exact IL-loading on P25 was determined by the thermogravimetric analysis (TGA, SDT650, TA Instrument) (Mirzaei et al., 2018; Mohamedali et al., 2019).

The ternary system of P25-[Hmim][NTf_2_]-squalane was carefully prepared using an electronic analytical balance (Sartorius SECURA225D−1CN) with a standard uncertainty of u(*m*) = 6.2 × 10^−5^ g. The five samples of P25-[Hmim][NTf_2_] with different IL-loading were used in the work.

### 2.3 Characterizations

The structure of [Hmim][NTf_2_] was identified with nuclear magnetic resonance spectroscopy (^1^H NMR, Bruker). The ^1^H NMR chemical shifts *δ* were as follows: 0.85 (t, 3H), 1.25 (s, 6H), 1.76 (q, 2H), 3.83 (s, 3H), 4.14 (t, 2H), 7.67–7.75 (d, 2H), 9.08 (s, 1H), which are consistent with those reported in the literature (Scharf et al., 2012; Kaviani et al., 2018). The results of ^1^H NMR are shown in Supplementary Figure S1. The crystal structure of the P25 sample was confirmed using the XRD patterns, as shown in Supplementary Figure S2. According to the XRD characterization, the diffraction peaks of P25 at 25.32°, 37.82°, 48.06°, 53.96°, 55.10°, 62.74°, 68.9°, 70.30°, and 75.14° are corresponding to the (101), (004), (200), (105), (211), (204), (116), (220), and (215) crystal planes of the anatase TiO_2_, while those at 27.44°, 36.10°, 41.26°, and 56.68° are the (110), (101), (111) and (220) facets of the rutile TiO_2_. The analysis indicated that P25 is a mixture of different crystalline phases of titanium dioxide, namely 81.1 wt% anatase phase and 18.9 wt% rutile phase. The intrinsic crystal structure of the P25-IL samples was confirmed using the XRD patterns for P25 and P25-IL. After loading [Hmim][NTf_2_], the diffraction pattern of P25 did not change, indicating that the structure of P25 did not change; while its diffraction peak intensity has a slight change, which is attributed to the influence of loading the IL on the P25 (Xie et al., 2016; Mohamedali et al., 2019).

Nitrogen adsorption/desorption isotherms were measured at 77 K to determine the surface area, total pore-volume, and average pore-size of the P25 and P25-IL samples through Micromeritics Tristar II 3020 analyzer (Micromeritics, United States). The specific surface area, total pore-volume, and average pore-size of P25 are 44.8 m^2^·g^−1^, 0.16 cm^3^·g^−1^, and 14.7 nm, respectively. As shown in Supplementary Figure S3, compared to the original P25, after the IL was loaded onto P25, the surface area and pore volume decreased dramatically. This result indicates that the IL loads onto the P25 surface (Harmanli et al., 2021). TGA was used to determine the exact IL-loading for P25-[Hmim][NTf_2_]. It was carried out under the N_2_ atmosphere, and the temperature was increased at a heating rate of 10 K·min^−1^ from room temperature to 973.2 K.

The solubility of [Hmim][NTf_2_] in squalane was characterized using Fourier transform infrared spectroscopy (FT-IR, NicoletiN10), a conductivity meter (Mettler-Toledo, S470K), and UV-vis spectroscopy (PerkinElmer Lambda 365). The conductivity measurements were accompanied by a relative standard uncertainty of u(*σ*) = 0.5%. In the measurements via UV-vis spectroscopy, solutions with different mass ratios of [Hmim][NTf_2_] to squalane were prepared, as shown in Table 2, and the prepared solutions were initially stirred strongly and allowed to reach the saturation equilibrium by separating these two phases for at least 72 h (Freire et al., 2008). The measurements were then conducted at 293.2 K.

**TABLE 2:** Mass ratios of [Hmim][NTf_2_] to squalane at 293.2 K and 0.1 MPa.

Samples No	*m*_IL_ (g)	*m*_squalane_ (g)	(*m*_IL_/*m*_squalane_) (g·g^−1^)
1	0.00735	100.000	7.35 × 10^−5^
2	0.01292	100.000	1.29 × 10^−4^
3	0.01712	100.000	1.71 × 10^−4^
4	0.02259	100.000	2.26 × 10^−4^
5	0.03371	100.000	3.37 × 10^−4^
6	0.05510	100.000	5.51 × 10^−4^

The temperature was controlled with an ultra-precision circulating water bath (C3100A) purchased from Xi’an Xiaxi Electronic Technology Co., Ltd. The temperature of the water bath was monitored by a high-precision platinum resistance thermometer (T1000/T6325, Xi’an Xiaxi Electronic Technology Co., Ltd). The standard uncertainty of temperature is u(*T*) = 0.02 K.

### 2.4 Reference state for interfacial ILs

In conventional solution thermodynamics, the asymmetric standard state is generally used, and the infinite dilution is used as the reference state. Following this, the standard thermodynamic properties of the solute reflect the interaction of the solute with the solvent (Akinfiev et al., 2016). Considering the infinite dilution in a conventional solution, for the ILs subject to interfacial interactions with solids, the reference state of the interfacial ILs can be defined as the one that the IL-film thickness tends to zero and the IL-moles at the interface also tends to zero, i.e., the IL-film thickness and the mole are the infinitesimal of the same order.

Here, assuming a uniform distribution and average spread of IL on the surface of P25, the film thickness of ILs (*δ*) on the surface of P25 can be estimated from the density of ILs, the loading weight of ILs and the specific surface area of immobilized ILs samples (Wu et al., 2017b) through Eqs 1, 2.

$$\delta =1000\times \frac{m_{P25-IL}\times wt\%}{m_{P25}\times S_{A}\times \rho_{IL}}$$ (1)

$$m_{P25-IL}=m_{P25}+m_{IL}$$ (2)

where *m*_P25-IL_, *m*_P25_, and *m*_IL_ are the mass amounts of P25-IL, P25, and IL, respectively, wt% is the mass fraction of IL, *S*_A_ is the specific surface area, and *ρ*_IL_ is the density of interfacial IL.

Moreover, the mole of ILs (*n*_IL_) on the surface of P25 is calculated by Eq. 3.

$$n_{IL}=\frac{m_{P25-IL}\times wt\%}{M_{IL}}$$ (3)

$$\lim_{\delta \to 0}\frac{\delta }{n}=\lim_{\delta \to 0}\frac{1000\times \frac{m_{P25-IL}\times wt\%}{m_{P25}\times S_{A}\times \rho_{IL}}}{\frac{m_{P25-IL}\times wt\%}{M_{IL}}}=\lim_{\delta \to 0}\frac{1000}{m_{P25}}\times \frac{M_{IL}}{S_{A}\times \rho_{IL}}=const$$ (4)

where *M*_IL_, *S*_A_, *ρ*_IL_, and *m*_P25_ all are constants, and, when *δ* tends to zero, *n*_IL_ or *m*_IL_ also tends to zero. Therefore, we say that *δ* and *n*_IL_ are infinitesimals of the same order, namely, as in Eq. 4.

### 2.5 ePC-SAFT-DFT modelling

In ePC-SAFT-DFT, the nine to three Lennard-Jones potential was used to represent the nonelectrostatic interaction between P25 and [Hmim][NTf_2_]. The formula of nine to three Lennard-Jones potential is shown below Eq. 5.

$$U_{s,9-3,i}\left(z\right)=\frac{2\pi \rho_{atom}\sigma_{si}^{3}\varepsilon_{si}}{3}\left[\frac{2}{15}{\left(\frac{\sigma_{si}}{z}\right)}^{9}-{\left(\frac{\sigma_{si}}{z}\right)}^{3}\right]$$ (5)

where *z* is the distance to the P25 surface, *ρ*_atom_ is the density of P25 (0.0293 Å^−3^), *σ*_si_ and *ε*_si_ are the size and energy parameters of P25-[Hmim][NTf_2_], which are determined by the Berthelot−Lorentz combining rules as below Eq. 6.

$$\left\{\begin{array}{c} \sigma_{si}=\frac{\sigma_{i}+\sigma_{s}}{2}\\ \varepsilon_{si}=\sqrt{\varepsilon_{i}\varepsilon_{s}} \end{array}\right.$$ (6)

The *σ*_i_ and *ε*_i_ represent the size and energy parameters of [Hmim][NTf_2_]. In this work, they were taken from the ePC-SAFT model fitted to the experimental data (*σ*_i_ = 3.7125 Å, *ε*_i_ = 2.44307 kJ·mol^−1^). *σ*_s_ and *ε*_s_ are the size and energy parameters of the solid surface, which were set as the adjustable parameters in this work. To assess the model performance, the average relative deviation (*ARD*) was calculated by the following Eq. 7

$$ARD=\frac{\left|\rho_{cal}-\rho_{\exp}\right|}{\rho_{\exp}}$$ (7)

where *ρ*_cal_ is the density of interfacial IL obtained by ePC-SAFT-DFT, and *ρ*_exp_ is the density of interfacial IL determined with the Gay-Lussac pycnometer.

## 3 Determination of density

The Anton Paar DMA 5000 densimeter, equipped with a temperature control system offering a temperature uncertainty of u(*T*) = 0.01 K, was used to measure the densities of [Hmim][NTf_2_], water, and squalane. The density measurements were conducted over a temperature ranging from 293.15 to 323.15 K, with a standard uncertainty of u(*ρ*) = 5 × 10^−6^ g·cm^-3^.

The Archimedes drainage method was used to determine the densities of P25 and P25-[Hmim][NTf_2_] through the pycnometers.

### 3.1 Densities of pure liquids

The densities of [Hmim][NTf_2_], squalane, and water were measured with Anton Paar DMA 5000 densimeter based on the “oscillating U-tube principle” operated in the static mode and automatically thermostated. Before the experiment, the densimeter was calibrated with dry air and ultrapure water. For the sake of improving the accuracy of the experiment, each sample was measured at least twice with a random error within the requirement of thermodynamic study, and the average value was reported.

### 3.2 Density determination with Gay-Lussac pycnometer

Pycnometers are usually flasks with a capillary neck (approximately 1 mm diameter) with a flat top. They are commonly used for density measurements of solids and different fluids, and the expanded uncertainty of density measurements was estimated to be 2 × 10^−4^ g·cm^−3^ (Lorefice et al., 2014). For the system with P25, their densities were determined with the Gay-Lussac pycnometer possessing extremely high accuracy. Since the experimental uncertainty strongly depends on the operating conditions, to ensure experimental reliability, intensive work was conducted from experimental preparation to practical operation.

Step 1: Experimental preparation and calibration. Before the experiment, the pycnometer was ultrasonically washed with acetone, ethanol, and ultrapure water in sequence until it was observed that the water on the inner wall neither aggregated into water droplets nor flowed down in streams. After drying for 8 h at 313.2 K in a blast drying oven, the pycnometer was taken out and placed in a laboratory drying oven for 24 h before starting the experiment. During the experiment, to ensure experimental accuracy, after cleaning the pycnometer, the weight change of the cleaned pycnometer compared with the original pycnometer was controlled to be less than 2 × 10^−4^ g.

The gravimetric method is the standard one used by accredited laboratories to calibrate volume instruments, such as pycnometers, including weighing the instrument both empty and full of pure water (or other liquid of known density). In this work, the ultrapure water was used to calibrate the volume of the pycnometer based on the process described as follows. The dry pycnometer was weighed with an analytical balance and recorded as *m*_1_. The degassed water was poured into the pycnometer with a syringe, and then heated and ultrasonically oscillated to remove the air bubbles. After that, the pycnometer filled with water was put into the water bath, keeping the temperature at 293.15 K for 30 min. During the process, the solvent was added or removed with a 10 μL micro-syringe under a constant temperature in a water bath. The pycnometer was taken out, and its outer wall was carefully wiped with the lens cleaning paper. After that, it was weighed and recorded as *m*_2_. Repeating the measurement at least 5 times in a cycle as described above to ensure the standard deviation satisfies the requirements of the thermodynamic study. The volume of the pycnometer was then determined by Eq. 8:

$$V_{pycnometer}=\frac{m_{2}-m_{1}}{\rho_{H_{2}O}}$$ (8)

where *ρ*_H_2_O_ is the density of water, and *V*_pycnometer_ is the volume of the pycnometer. The high borosilicate glass pycnometer used in this work has a low coefficient of thermal expansion of 3.3 × 10^−6^ K^−1^ (Lima and Monteiro, 2001). The volume change between 293.15 and 323.15 K is only 3 × 10^−4^ cm^3^, which is negligible compared to the volume detected in this work. Therefore, in this work, a volume calibration at 293.15 K is sufficient.

Step 2: Measuring the density of water (*ρ*_H_2_O_). Before determining the density of samples, the density measurement of water was performed to verify the accuracy of the density determination of the pycnometer. The process of determining the density of water is the same as the process of calibrating the pycnometer.
Step 3: Determining the density of P25 (*ρ*_P25_). Firstly, the empty pycnometer was weighted with the balance and recorded as *m*_3_; secondly, a certain amount of P25 particles was added, and the total weight of the pycnometer and P25 was recorded as *m*_4_; finally, a 10 μL micro-syringe was used to add solvent (water or squalane in this work) until the entire pycnometer was filled. The pycnometer with the samples was kept in the water bath for 30 min and then taken out and wiped off the solvent outside with the lens cleaning paper. The total weight was determined and recorded as *m*_5_. Generally, there is no excess volume between solid particles and solvent, and thus the density of P25 (*ρ*_P25_) can be determined by Eq. 9a and Eq. 9b:

$$\rho_{P25}=\frac{m_{4}-m_{3}}{V_{1-pycnometer}-\frac{m_{5}-m_{4}}{\rho_{solvent}}}$$ (9a)

$$\rho_{P25}=\frac{\left(m_{4}-m_{3}\right)\times \rho_{solvent}}{\left(m_{2}-m_{1}\right)-\left(m_{5}-m_{4}\right)}$$ (9b)

Samples were measured five times after each addition or removal of solvent. The average value was reported.

Step 4: Determining the density of P25-[Hmim][NTf_2_] (*ρ*_P25-IL_). In this work, squalane was chosen as the solvent, which is physicochemically stable over a wide temperature range (Choi et al., 2009) with low vapor pressure (Dubey and Sharma, 2008a) and insoluble with water (to avoid the containment from the water bath and air humidity). Particularly, [Hmim][NTf_2_] is insoluble in squalane; it will thus not affect the properties of P25-[Hmim][NTf_2_], and the excess volume of mixing can be ignored.

The experimental method for determining the density of P25-[Hmim][NTf_2_] followed that for P25, and the only difference is on the sample constituent. Here, P25-[Hmim][NTf_2_] was used as solid particles, and squalane was used as the solvent. The density of P25-[Hmim][NTf_2_] can be determined by Eq.10a and Eq. 10b:

$$\rho_{P25-IL}=\frac{m_{7}-m_{6}}{V_{pycnometer}-\frac{m_{8}-m_{7}}{\rho_{squalane}}}$$ (10a)

$$\rho_{P25-IL}=\frac{m_{7}-m_{6}}{\frac{m_{2}-m_{1}}{\rho_{H_{2}O}}-\frac{m_{8}-m_{7}}{\rho_{squalane}}}$$ (10b)

where *m*_6_ represents the weight of the empty pycnometer, *m*_7_ is that for the pycnometer and P25-[Hmim][NTf_2_], and *m*_8_ refers to the total weight of the pycnometer and P25-[Hmim][NTf_2_]-squalane.

## 4 Results and discussion

### 4.1 Densities of [Hmim][NTf_2_], squalane, and water

**FIGURE 1:**
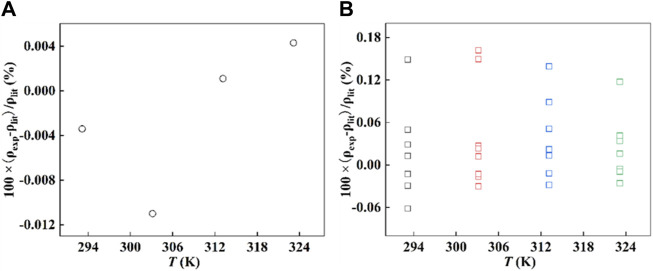
The deviation between the experimental density, *ρ*_exp_, and the density collected from the literature (Kuss and Taslimi, 1970; Graaf et al., 1992; Kumagai and Takahashi, 1995; Fandin et al., 2005; Kumagai et al., 2006; Dubey and Sharma, 2008b; Dubey and Sharma, 2009a; Dubey and Sharma, 2009b; Harris, 2009; Fandiño et al., 2010; Korotkovskii et al., 2012; Paredes et al., 2020; Bürk et al., 2021; Yang et al., 2021), *ρ*_lit_. **(A)** [Hmim][NTf_2_] and **(B)** squalane, (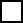) 293.15 K, (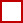) 303.15 K, (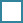) 313.15 K, and (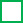) 323.15 K.

The densities of [Hmim][NTf_2_], squalane, and water were measured with the Anton Paar DMA 5000 densimeter at 293.15, 303.15, 313.15, and 323.15 K, and each sample was repeated at least twice. The experimental data are in Supplementary Table S1. The densities of [Hmim][NTf_2_] measured in this work were also compared with those reported in the recommended literature (Paredes et al., 2020), and, as shown in Figure 1A, the relative error was less than 0.012%. In addition, the deviation of squalane with purity essentially above 99 wt% is also less than 0.2%, as shown in Figure 1B. In summary, the comparison shows that the values determined in this work are in good agreement with those reported values, and they are reliable.

### 4.2 Determination of densities of P25 and P25-[Hmim][NTf_2_]

This work aims to determine the densities or molar volumes of immobilized ILs that depends on the IL-loading and based on the assumption that [Hmim][NTf_2_] is completely insoluble in squalane for the studied case. Therefore, in this part, besides the density measurements, the IL-loading and the solubility of [Hmim][NTf_2_] in squalane were also investigated.

#### 4.2.1 Determination of IL-loading

**FIGURE 2:**
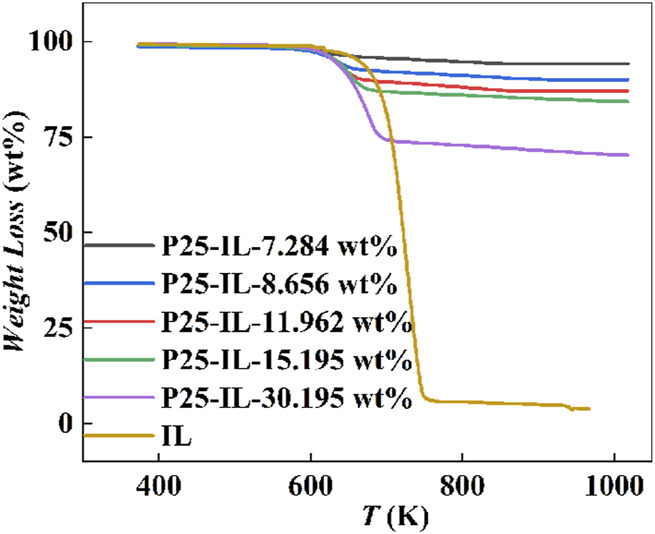
TGA curves of [Hmim][NTf_2_] and P25-[Hmim][NTf_2_].

The characteristic thermal decomposition curves of [Hmim][NTf_2_] and P25-[Hmim][NTf_2_] are illustrated in Figure 2, showing that the weight loss of [Hmim][NTf_2_] mainly occurs in the range of 100–700 °C. Based on the weight loss, the real IL-loadings were estimated to be 7.284, 8.656, 11.962, 15.195, and 30.195 wt%, respectively, for the studied five P25-[Hmim][NTf_2_] samples. Further analysis of the thermal stability of the ILs on the surface of P25 was conducted, with the thermogravimetric curves of the pure and supported ILs being analyzed. The results demonstrated that the decomposition temperatures of the ILs increased with increasing IL loadings. The decomposition temperatures of the ILs were found to be 325.20, 330.95, 338.63, 348.82, and 366.10°C, respectively. In contrast, the decomposition temperature of the pure ILs was found to be 416.97 ^o^C. This indicates that, with an increase in IL loadings, the IL far away from the surface is gradually weakened by the interaction with the surface, approaching the nature of bulk ILs. According to the interpretation of the hinged spring model proposed by Singh (Singh et al., 2014), the imidazolium ring has been postulated to be ‘hinged’ to the P25 through surface oxygen interacting with the C-H groups of the imidazole ring, while the corresponding alkyl chains are in a free state. When the sample is heated, the alkyl chains on the cation are more likely to detach, leading to the eventual detachment of the imidazole ring. Consequently, ILs affected by the interface appear to lower the decomposition temperature (Verma et al., 2020; Wu et al., 2020).

#### 4.2.2 Solubility of [Hmim][NTf_2_] in squalane

The insolubilization of [Hmim][NTf_2_] in squalane is a prerequisite for determining the density of P25-[Hmim][NTf_2_] in squalane. Therefore, UV-vis, FT-IR, and conductivity experiments were performed in this part.

**FIGURE 3:**
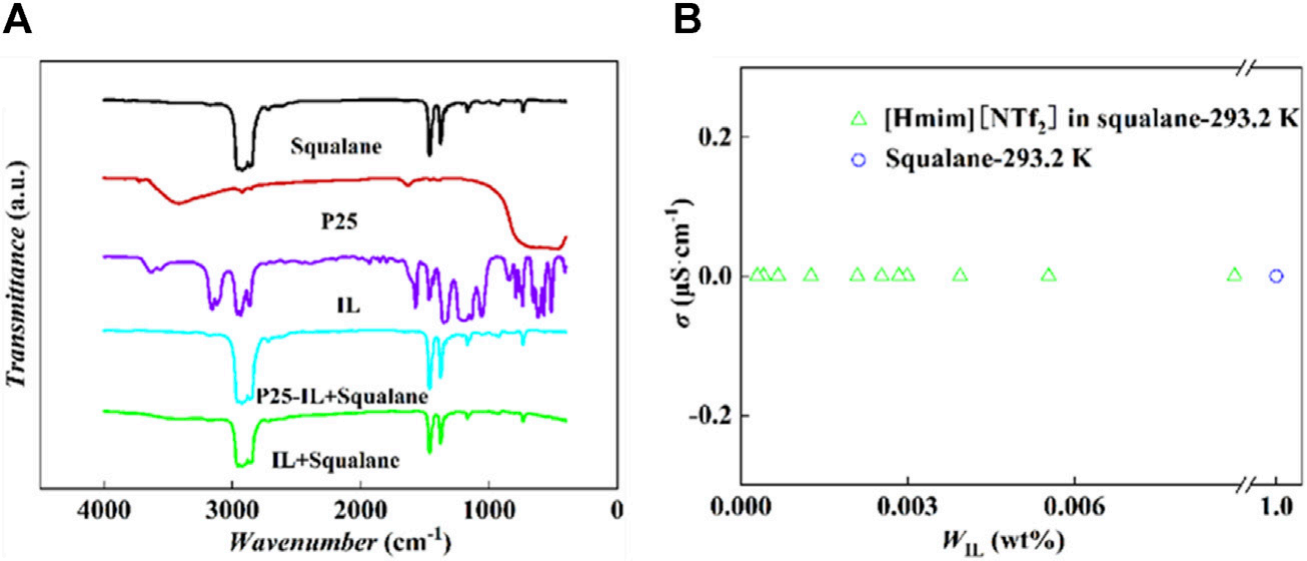
Characterization of the insolubilization of [Hmim][NTf_2_] in squalane. **(A)** FT-IR spectra. **(B)** Conductivity experiments: [Hmim][NTf_2_] in squalane at 293.2 K.

The FT-IR spectra for pure squalane, P25, and IL were detected. Meanwhile, the upper solutions of the binary (IL-squalane) and ternary (P25-IL-squalane) systems were also sampled for the infrared analysis. As shown in Figure 3A, the infrared peaks of the supernatants from both the binary (IL-squalane) and ternary (P25-IL-squalane) systems are completely the same as those for pure squalane, and there is no characteristic peak of IL and P25. Therefore, neither IL in the ternary system was drawn off from the interface and dissolved in squalane, nor IL in the binary system was in squalane. Therefore, [Hmim][NTf_2_] is completely insoluble in squalane. Further comparison of the FT-IR spectra of squalane and the supernatants of the ternary (P25-IL-squalane) system with five different IL loading amounts shows that no characteristic peaks of ILs and P25 were observed. The related characterization results are shown in Supplementary Figure S4.

To further confirm the insolubilization, the conductivity analysis was performed with the method proposed by Karel (Rehak et al., 2012), where the conductivities of the upper solutions from a series of [Hmim][NTf_2_]-squalane mixtures were determined at 293.2 K. Based on the results shown in Figure 3B, the conductivity of the upper solution from this series of [Hmim][NTf_2_]-squalane with different IL fractions is almost a constant, which is exactly the same as the pure squalane (i.e., 0 mS·cm^−1^). This indicates that no anions or cations are dissolved in squalane.

Additionally, the UV-vis spectrophotometer was used to quantitatively study the solubility of ILs in squalane. In the measurements, firstly, the upper solution of the mixture was scanned in wavelengths ranging from 200 to 1,000 nm. The maximum absorption wavelength was obtained at 292 nm, as shown in Supplementary Figure S5A. Secondly, the absorbance of the mixture with different mass ratios at 292 nm wavelength is illustrated in Supplementary Figure S5B, showing that, as the mass ratio of [Hmim][NTf_2_] to squalane decreases from 5.51 × 10^−4^ to 7.35 × 10^−5^, the corresponding absorbance value remains unchanged. This observation suggests that the solubility of [Hmim][NTf_2_] in squalane is very low, which can be ignored in the experimental determination of interfacial IL density.

Combining the infrared spectroscopy, conductivity experiments, and UV-vis spectrophotometer detections, we can conclude that [Hmim][NTf_2_] is insoluble in squalane, and subsequently, their excess volume can be neglected.

#### 4.2.3 Densities of P25 and P25-[Hmim][NTf_2_]

Six pycnometers were used in the density determination in this work. Their volumes were calibrated with the ultrapure water and then estimated with Eq. 1. Their specific volumes are 25.4640, 9.9926, 10.2967, 10.4339, 10.5762, and 8.6258 cm^3^, respectively, and their standard deviations in measurements are 0.016%, 0.036%, 0.023%, 0.018%, 0.018%, and 0.036%. The original experimental data and error analysis are listed in Supplementary Tables S2–S7, Supplementary Tables S16, S17.

At 0.1 MPa and 293.15 K, a pycnometer with a calibrated volume was randomly used to measure the density of ultrapure water to determine the accuracy of the pycnometer. The average of the five experimental measurements is 0.998 g·cm^-3^. The density of water determined by the pycnometer is in line with the literature when compared with the literature values, and the relative errors are 0.01%, satisfying the thermodynamic error requirements. The experimental results can be seen in Supplementary Table S8.

The density of P25 was measured with the drainage method using the pycnometers with the precisely determined volume. Here both H_2_O and squalane were used as the solvent. The original experimental data points and error analysis for determining the density of P25 are listed in Supplementary Table S9, S10 and Supplementary Table S18. The density of P25 was calculated with Eq. 6. The result is 3.681 g·cm^−3^ with an expanded uncertainty of 0.008 g·cm^−3^ (*k* = 2, 95% confidence level) when using water as the solvent, and it is 3.681 g·cm^−3^ with an expanded uncertainty of 0.007 g·cm^−3^ (*k* = 2, 95% confidence level) when using squalane as the solvent. Based on these measurements, the deviation of the density of P25, whether using squalane or water as the solvent, falls within the range of experimental uncertainty, indicating that both two solvents can be used in measuring the density of P25. According to the determined density using water as the solvent, the volume of P25 is 0.749 cm^3^, and the expanded uncertainty is 0.003 cm^3^ (*k* = 2, 95% confidence level).

Similarly, the density of P25-[Hmim][NTf_2_] was determined, where the samples of P25-[Hmim][NTf_2_] with different IL mass fractions (i.e., IL-loadings) were used. In the preparation, the weight of P25 for each sample was kept the same, and the mass fractions of IL were 7.284, 8.656, 11.962, 15.195, and 30.195 wt%, respectively. The determined results are listed in Supplementary Tables S11–S15 for different samples, and their standard deviations are 0.059%, 0.041%, 0.041%, 0.036%, and 0.022%, respectively. The analysis of experimental uncertainty and repeatability in detail is described in Supplementary Table S19.

### 4.3 Standard molar volume of immobilized [Hmim][NTf_2_] ( $V_{\text{interface}‐\text{IL}}^{\infty }$ )

The standard molar volume of immobilized IL was obtained based on the principle of colligative properties by using the data determined with the pycnometer. Briefly, (1) *n*_IL_, the volume of squalane (*V*_squalane_), and the volume of [Hmim][NTf_2_] at the interface (*V*_interface-IL_) were calculated; (2) the standard molar volume of immobilized IL ( $V_{\text{interface}‐\text{IL}}^{\infty }$ ) was then obtained via linear extrapolation, which is a standard method for obtaining the thermodynamic properties. A more specific description was described below. The analysis of experimental uncertainty and repeatability in detail is described in Supplementary Table S20, S21.

Step 1, obtaining *n*_IL_. Firstly, the weight of P25-[Hmim][NTf_2_] in the pycnometer was determined, and the weight of IL was obtained according to its mass fraction determined by TGA. Then, *n*_IL_ was calculated based on the molar mass of [Hmim][NTf_2_] (*M*_IL_ = 447.41 g·mol^−1^). The corresponding results are listed in Table 3.
Step 2, determining *V*_squalane_. *V*_squalane_ added into the pycnometer was determined from its weight and the density measured by the Anton Paar densitometer, according to Eq. 11. The corresponding results are listed in Table 3.

$$V_{squalane}=\frac{m_{squalane}}{\rho_{squalane}}$$ (11)

Step 3, estimating *V*_interface-IL_. According to the above discussion and analysis, it is reasonable to assume that the volume of pycnometer is equal to the sum of (1) the volume of IL at the interface, (2) the volume of squalane, and (3) the volume of P25. Subsequently, the volume of IL (i.e., [Hmim][NTf_2_]) at the interface in the pycnometer can be calculated with Eq. 12. The results are listed in Table 3.

$$V_{interface-IL}=V_{pycnometer}-V_{P25}-V_{squalane}$$ (12)

**TABLE 3:** IL-Loading for P25-[Hmim][NTf_2_], *n*_IL_, *V*_squalane_, and *V*_interface-IL_ at 293.15 K and 0.1 MPa.

P25-IL (wt%)	10^4^ × *n*_IL_ (mol)	*V*_squalane_ (cm^3^)	*V*_interface-IL_ (cm^3^)
7.284 8.656 11.962 15.195 30.195	4.84 5.84 8.38 11.05 26.67	9.4134 9.5210 9.5806 7.5337 8.6970	0.134 0.164 0.246 0.343 0.850

**FIGURE 4:**
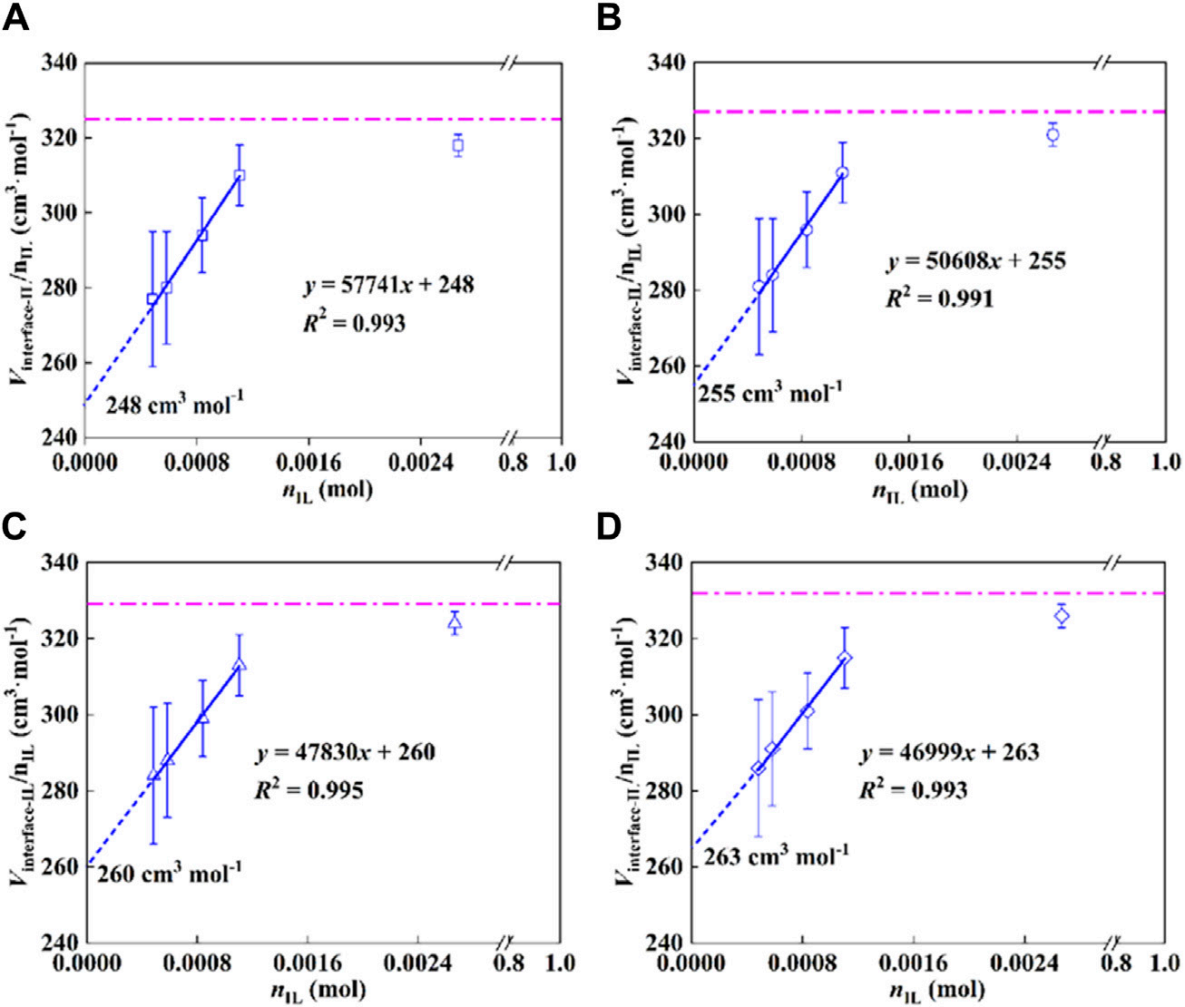
Molar volume of IL at the P25 interface as a function of moles of IL, **(A)** 293.15 K, **(B)** 303.15 K, **(C)** 313.15 K, and **(D)** 323.15 K.

According to the determined density for each sample with different IL-loadings, the molar volume of IL at the interface (*V*_m-interface-IL_ = *V*_interface-IL_/*n*_IL_) was calculated. The results are shown in Figure 4, the molar volume of the interfacial IL showed a linear relationship with the moles of the IL, satisfying the colligative law of solution, and a standard molar volume was obtained by taking the limit. More specifically, when the moles of ILs at the interface approach zero, the standard molar volume of the immobilized IL is obtained by a linear extrapolation, as expressed in Eq. 13.

$$V_{m-interface-IL}^{\infty }=\lim_{n_{IL}\to 0}\frac{V_{m-interface-IL}}{n_{IL}}$$ (13)

As shown in Figure 4, the standard molar volumes of the immobilized ILs are 248, 255, 260, and 263 cm^3^·mol^−1^ at temperatures of 293.15, 303.15, 313.15, and 323.15 K, respectively. The expanded uncertainty of the standard molar volume of the interfacial ILs is 18 cm^3^·mol^−1^ (*k* = 2, confidence level 0.95). Meanwhile, the dashed lines in the figure represent the molar volumes of the bulk ILs with the values 325, 327, 329, and 332 cm^3^·mol^−1^ at 293.15, 303.15, 313.15, and 323.15 K. Compared with the bulk IL, its molar volume decreased by 23.7%, 22.0%, 21.0%, and 20.8%, correspondingly. The results imply that the state of IL immobilized on the substrate is in a compressed state, that is, the existence of the interface changes the molar volume of the original bulk IL, which makes the change of the IL structure and the arrangement becomes more compact. Molecular simulations (Wang et al., 2014; Kritikos et al., 2016) and experimental studies (Bañuelos et al., 2013) reported in the literature show that the strong interaction at the interface makes the arrangement of ILs more ordered (Herrera et al., 2015; Zhang et al., 2017).

**FIGURE 5:**
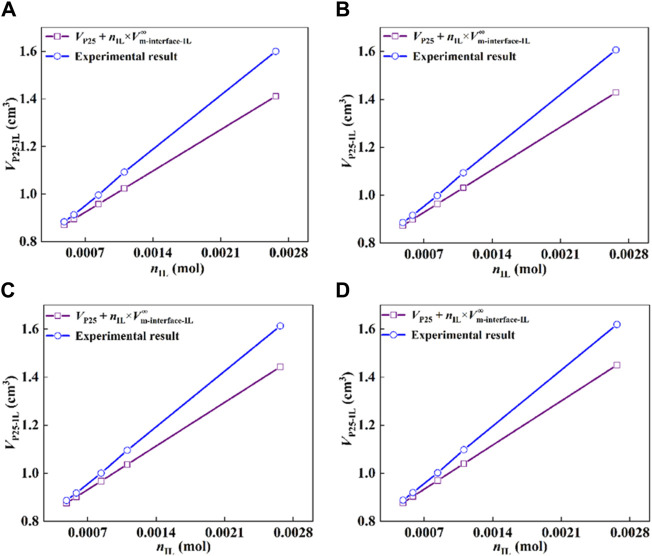
Comparison of the volume of P25-IL determined experimentally with the ideal mixing results based on the standard molar volumes of IL and P25, **(A)** 293.15 K, **(B)** 303.15 K, **(C)** 313.15 K, and **(D)** 323.15 K.

The results at different temperatures are shown in Figure 5. The comparison of the volume obtained by an ideal summation of the standard molar volume of [Hmim][NTf_2_] and P25 with the experimental results shows consistent results for the systems with an IL-film thickness on the surface of P25 over an infinitely narrow range of films. As the film thickness increases, the calculated results deviate increasingly from the experimental results, indicating that the film thickness region no longer fits the calculated results.

**FIGURE 6:**
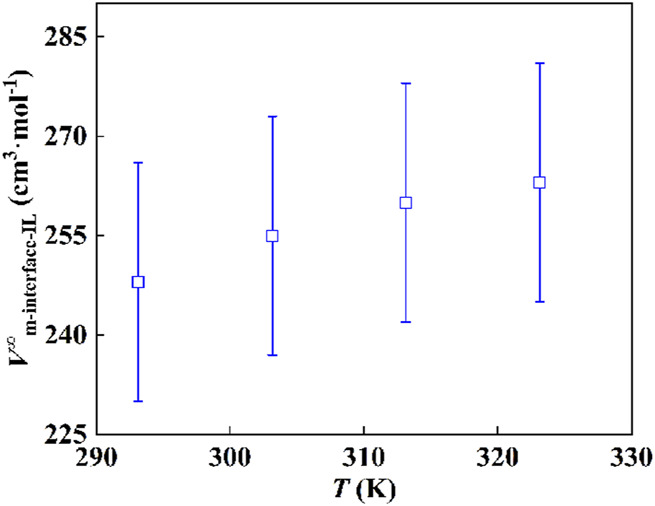
The standard molar volume of the IL on the interface varies with temperature.

The standard molar volumes of interfacial ILs at different temperatures were obtained by the solution colligative property. Subsequently, the variation of the standard molar volume with temperature was plotted. As shown in Figure 6, as the temperature increases, the standard molar volume of the IL at the interface increases. In addition, as the temperature increases, the increasing trend of the standard molar volume gradually slows down. We think that the IL layer near the interface is relatively stable, and the influence of the temperature increase gradually decreases.

### 4.4 ePC-SAFT-DFT modelling

**FIGURE 7:**
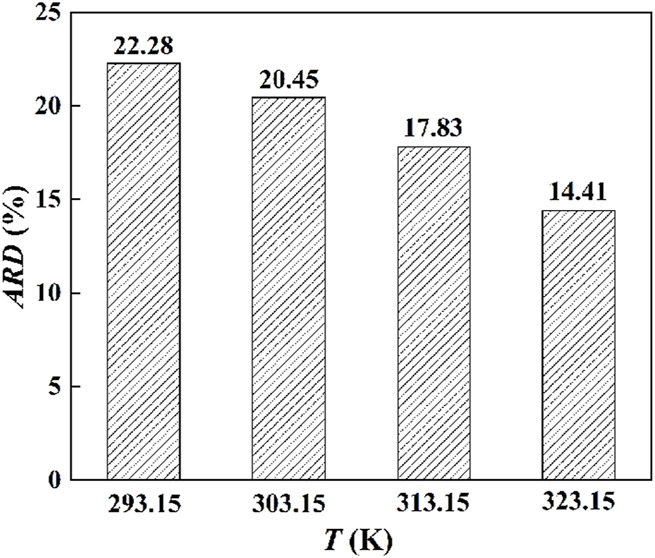
The *ARD* of ePC-SAFT-DFT with the parameters from molecular simulations (*σ*_s_ = 3.475 Å and *ε*_s_ = 0.29346 kJ·mol^−1^).

Based on the standard molar volume of [Hmim][NTf_2_] on the P25 surface determined in this work, the model parameters, *σ*_s_ and *ε*_s_, of ePC-SAFT-DFT were obtained. The fitted values and the corresponding *ARD* at different temperatures are listed in Table 4, where the average *ARD* is only 0.68%. In molecular simulations, *σ*_s_ = 3.475 Å and *ε*_s_ = 0.29346 kJ·mol^−1^ were used. For comparison, this set of parameters were used to predict the density of [Hmim][NTf_2_] on the P25 surface, and the corresponding *ARD* at different temperatures is higher than 10%, as shown in Figure 7. This indicates the importance of determining experimental data in the development of thermodynamic models in order to obtain the model parameters.

**TABLE 4:** The fitted parameters of ePC-SAFT-DFT and the corresponding *ARD* at different temperatures.

*T* (K)	*σ*_s_ (Å)	*ε*_s_ (kJ·mol^−1^)	*ARD* (%)
293.15	2.846	0.269	0.52
303.15	3.012	0.249	1.13
313.15	3.168	0.233	0.99
323.15	3.210	0.240	0.09

**FIGURE 8:**
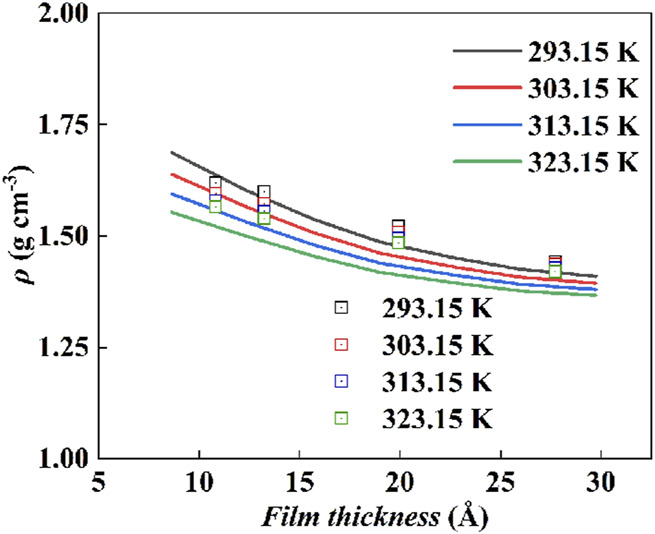
The comparison of ePC-SAFT-DFT predictions with the experiment values for the density of IL with varying film thickness, solid line: model prediction; square box: experimental values.

Figure 8 shows the comparison, where the ePC-SAFT-DFT model with the determined parameters was used to predict the densities of the [Hmim][NTf_2_] at the P25 interface at different film thicknesses, with the experimental values. Obviously, the model predictions at 293.15, 303.15, 313.15, and 323.15 K are in good agreement with the experimental results, and their *ARD*s are 1.67%, 2.16%, 2.84%, and 3.69%, respectively. As shown in Figure 8, the accuracy of the model predictions increases as the film thickness of the IL decreases, also suggesting that the obtained interfacial standard thermodynamic properties are reliable.

## 5 Conclusion

In this work, a new method was proposed and developed to determine the standard molar volume of immobilized IL, based on the combination of the Archimedes drainage method and the colligative law, together with the ingenious experimental design and solvent selection. The [Hmim][NTf_2_] immobilized on P25 was used as one example to demonstrate the method. It was found that the standard molar volumes of [Hmim][NTf_2_] immobilized on P25 were 248, 255, 260, and 263 cm^3^·mol^−1^ at temperatures of 293.15, 303.15, 313.15, and 323.15 K, respectively. The expanded uncertainty of the standard molar volumes of the interfacial ILs is 18 cm^3^·mol^−1^ (*k* = 2, confidence level 0.95). Compared with the IL in the bulk phase, the standard molar volume of the immobilized IL decreased by 23.7%, 22.0%, 21.0%, and 20.8%, respectively, indicating that the immobilized IL is in a compressed state. The newly determined standard molar volume of [Hmim][NTf_2_] on P25 surfaces can be used to parameterize the ePC-SAFT-DFT model to further predict the densities of interfacial ILs with varying film thickness reliably. This work, for the first time, demonstrates the determination of standard thermodynamic properties for the immobilized ILs experimentally, and it also provides a basis for establishing a thermodynamic model of interfacial fluids.

## Data Availability

The original contributions presented in the study are included in the article/Supplementary Material, further inquiries can be directed to the corresponding authors.
