# Supplementary material for: Determination of standard molar volume of 1-hexyl-3-methylimidazolium bis(trifluoromethylsulfonyl)imide on titanium dioxide surface
Source: Front Chem. 2024 Jun 20;12:1416294. doi: 10.3389/fchem.2024.1416294 (PMC11225411; doi:10.3389/fchem.2024.1416294)
Supplement: Supplementary file 1 [file DataSheet1.docx]

Supplementary Material

# Supplementary Data

Table S1 The density of [Hmim][NTf_2_], squalane, and H_2_O at 293.15 K under 0.1 MPa.

|  | *T* (^o^C) | *ρ* (g·cm^-3^) | *T* (^o^C) | *ρ* (g·cm^-3^) | Average *ρ*(g·cm^-3^) |
| --- | --- | --- | --- | --- | --- |
| [Hmim][NTf_2_] | 19.99  30.00  40.00  50.00 | 1.376441  1.367240  1.358085  1.348998 | 19.99  30.00  40.00  50.00 | 1.376545  1.367245  1.358090  1.349002 | 1.376493  1.367243  1.358088  1.349000 |
| Squalane | 20.00  30.00  40.01  50.01 | 0.808504  0.802099  0.795705  0.789327 | 19.99  30.01  40.01  50.01 | 0.808501  0.802098  0.795707  0.789326 | 0.808503  0.802099  0.795706  0.789327 |
| H_2_O | 19.99 | 0.998206 | 20.00 | 0.998205 | 0.998206 |
| The standard uncertainty: *u*(*T*)= 0.01 K, *u*(*ρ*)= 5×10^−6^ g·cm^-3^, *u*(*P*)= (0~10) Pa | | | | | |

Table S2 Pycnometer volume calibration experiment data for measuring P25 density, *m*_1_: pycnometer weight, *m*_2_: weight of pycnometer filled with ultrapure water.

| *m*_1_ (g) | *m*_2_ (g) | *T* (^o^C) | *ρ*_H2O_ (g·cm^-3^) | *V*_pycnometer_ (cm^3^) | Average *V*_pycnometer_ (cm^3^) | |
| --- | --- | --- | --- | --- | --- | --- |
| 17.62510 | 43.04344 | 19.99 | 0.998208 | 25.4640 |  | |
| 17.62510 | 43.04359 | 20.00 | 0.998206 | 25.4642 |  | |
| 17.62509 | 43.04325 | 20.00 | 0.998206 | 25.4648 | 25.4640 | |
| 17.62507 | 43.04315 | 20.00 | 0.998206 | 25.4638 |  | |
| 17.62509 | 43.04336 | 20.00 | 0.998206 | 25.4640 |  | |
| The standard uncertainty: *u*(*m*_1_)= 6.3×10^-5^ g, *u*(*m*_2_)= 1.3×10^-4^ g, *u*(*T*)= 0.02 K, *u*(*ρ*_H2O_)= 5×10^-6^ g·cm^-3^. The expanded uncertainty (*k* = 2, 95% confidence level): *U*_c_(*V*_pycnometer_)= 4×10^-4^ cm^3^. | | | | | |  |

Table S3 Pycnometer volume calibration experiment data for measuring P25 density, *m*_1_: pycnometer weight, *m*_2_: weight of pycnometer filled with ultrapure water.

| *m*_1_ (g) | *m*_2_ (g) | *T* (^o^C) | *ρ*_H2O_ (g·cm^-3^) | *V*_pycnometer_ (cm^3^) | Average *V*_pycnometer_ (cm^3^) | |
| --- | --- | --- | --- | --- | --- | --- |
| 16.45096 | 26.42579 | 20.00 | 0.998206 | 9.9928 |  | |
| 16.45094 | 26.42521 | 20.00 | 0.998206 | 9.9922 |  | |
| 16.45099 | 26.42573 | 20.00 | 0.998206 | 9.9927 | 9.9926 | |
| 16.45095 | 26.42517 | 20.00 | 0.998206 | 9.9931 |  | |
| 16.45096 | 26.42561 | 20.00 | 0.998206 | 9.9921 |  | |
| The standard uncertainty: *u*(*m*_1_)= 6.3×10^-5^ g, *u*(*m*_2_)= 1.3×10^-4^ g, *u*(*T*)= 0.02 K, *u*(*ρ*_H2O_)= 5×10^-6^ g·cm^-3^. The expanded uncertainty (*k* = 2, 95% confidence level): *U*_c_(*V*_pycnometer_)= 4×10^-4^ cm^3^. | | | | | |  |

Table S4 Pycnometer volume calibration experiment data for measuring P25-[Hmim][NTf_2_]-squalane density, *m*_1_: pycnometer weight, *m*_2_: weight of pycnometer filled with ultrapure water.

| *m*_1_ (g) | *m*_2_ (g) | *T* (^o^C) | *ρ*_H2O_ (g·cm^-3^) | *V*_pycnometer_ (cm^3^) | Average *V*_pycnometer_ (cm^3^) | |
| --- | --- | --- | --- | --- | --- | --- |
| 11.29752 | 21.57538 | 20.00 | 0.998206 | 10.2963 |  | |
| 11.29752 | 21.57578 | 20.00 | 0.998206 | 10.2967 |  | |
| 11.29756 | 21.57574 | 20.00 | 0.998206 | 10.2967 | 10.2967 | |
| 11.29752 | 21.57592 | 20.00 | 0.998206 | 10.2969 |  | |
| 11.29753 | 21.57595 | 20.00 | 0.998206 | 10.2969 |  | |
| The standard uncertainty: *u*(*m*_1_)= 6.3×10^-5^ g, *u*(*m*_2_)= 1.5×10^-4^ g, *u*(*T*)= 0.02 K, *u*(*ρ*_H2O_)= 5×10^-6^ g·cm^-3^. The expanded uncertainty (*k* = 2, 95% confidence level): *U*_c_(*V*_pycnometer_)= 3×10^-4^ cm^3^. | | | | | |  |

Table S5 Pycnometer volume calibration experiment data for measuring P25-[Hmim][NTf_2_]-squalane density, *m*_1_: pycnometer weight, *m*_2_: weight of pycnometer filled with ultrapure water.

| *m*_1_ (g) | *m*_2_ (g) | *T* (^o^C) | *ρ*_H2O_ (g·cm^-3^) | *V*_pycnometer_ (cm^3^) | Average *V*_pycnometer_ (cm^3^) | |
| --- | --- | --- | --- | --- | --- | --- |
| 10.93730 | 21.35252 | 20.00 | 0.998206 | 10.4339 |  | |
| 10.93731 | 21.35219 | 20.00 | 0.998206 | 10.4336 |  | |
| 10.93733 | 21.35271 | 20.00 | 0.998206 | 10.4341 | 10.4339 | |
| 10.93733 | 21.35255 | 20.00 | 0.998206 | 10.4339 |  | |
| 10.93734 | 21.35257 | 20.00 | 0.998206 | 10.4340 |  | |
| The standard uncertainty: *u*(*m*_1_)= 6.2×10^-5^ g, *u*(*m*_2_)= 1.2×10^-4^ g, *u*(*T*)= 0.02 K, *u*(*ρ*_H2O_)= 5×10^-6^ g·cm^-3^. The expanded uncertainty (*k* = 2, 95% confidence level): *U*_c_(*V*_pycnometer_)= 3×10^-4^ cm^3^. | | | | | |  |

Table S6 Pycnometer volume calibration experiment data for measuring P25-[Hmim][NTf_2_]-squalane density, *m*_1_: pycnometer weight, *m*_2_: weight of pycnometer filled with ultrapure water.

| *m*_1_ (g) | *m*_2_ (g) | *T* (^o^C) | *ρ*_H2O_ (g·cm^-3^) | *V*_pycnometer_ (cm^3^) | Average *V*_pycnometer_ (cm^3^) | |
| --- | --- | --- | --- | --- | --- | --- |
| 10.59097 | 21.14836 | 20.01 | 0.998204 | 10.5764 |  | |
| 10.59102 | 21.14851 | 20.01 | 0.998204 | 10.5765 |  | |
| 10.59097 | 21.14804 | 20.00 | 0.998206 | 10.5761 | 10.5762 | |
| 10.59103 | 21.14822 | 20.00 | 0.998206 | 10.5762 |  | |
| 10.59100 | 21.14815 | 20.00 | 0.998206 | 10.5761 |  | |
| The standard uncertainty: *u*(*m*_1_)= 6.2×10^-5^ g, *u*(*m*_2_)= 1.2×10^-4^ g, *u*(*T*)= 0.02 K, *u*(*ρ*_H2O_)= 5×10^-6^ g·cm^-3^. The expanded uncertainty (*k* = 2, 95% confidence level): *U*_c_(*V*_pycnometer_)= 3×10^-4^ cm^3^. | | | | | |  |

Table S7 Pycnometer volume calibration experiment data for measuring P25-[Hmim][NTf_2_]-squalane density, *m*_1_: pycnometer weight, *m*_2_: weight of pycnometer filled with ultrapure water.

| *m*_1_ (g) | *m*_2_ (g) | *T* (^o^C) | *ρ*_H2O_ (g·cm^-3^) | *V*_pycnometer_ (cm^3^) | Average *V*_pycnometer_ (cm^3^) |  |
| --- | --- | --- | --- | --- | --- | --- |
| 14.87802 | 23.48792 | 20.00 | 0.998206 | 8.6254 |  |  |
| 14.87804 | 23.48866 | 20.00 | 0.998206 | 8.6261 |  |  |
| 14.87805 | 23.48800 | 20.00 | 0.998206 | 8.6254 | 8.6258 |  |
| 14.87809 | 23.48872 | 20.00 | 0.998206 | 8.6261 |  |  |
| 14.87805 | 23.48853 | 20.00 | 0.998206 | 8.6260 |  |  |
| The standard uncertainty: *u*(*m*_1_)= 6.3×10^-5^ g, *u*(*m*_2_)= 2.2×10^-4^ g, *u*(*T*)= 0.02 K, u(*ρ*_H2O_)= 5×10^-6^ g·cm^-3^. The expanded uncertainty (*k* = 2, 95% confidence level): *U*_c_(*V*_pycnometer_)= 5×10^-4^ cm^3^. | | | | | | |

Table S8 Determine the density of water.

| *m*_pycnometer_ (g) | *m*_pycnometer/H2O_ (g) | *T* (^o^C) | *V*_pycnometer_ (cm^3^) | *ρ*_H2O_ (g·cm^-3^) | Average *ρ*_H2O_ (g·cm^-3^) |  |
| --- | --- | --- | --- | --- | --- | --- |
| 10.59111 | 21.14737 | 19.99 |  | 0.9981 |  |  |
| 10.59112 | 21.14774 | 20.00 |  | 0.9982 |  |  |
| 10.59112 | 21.14732 | 20.00 | 10.5762 | 0.9981 | 0.9981 |  |
| 10.59109 | 21.14785 | 20.01 |  | 0.9982 |  |  |
| 10.59108 | 21.14753 | 20.00 |  | 0.9981 |  |  |
| The standard uncertainty: *u*(*m*_pycnometer_)= 6.2×10^-5^ g, *u*(*m*_pycnometer/H2O_)= 1.2×10^-4^ g, *u*(*T*)= 0.02 K. The expanded uncertainty (*k* = 2, 95% confidence level): *U*_c_(*ρ*_H2O_)= 4×10^-4^ g·cm^-3^. | | | | | | |

Table S9 Experimental data for the determination of P25 density using water as the solvent. *m*_3_: pycnometer weight, *m*_4_: weight of pycnometer plus P25, *m*_5_: weight of pycnometer plus sample plus full bottle of water.

| *m*_3_ (g) | *m*_4_ (g) | *m*_5_ (g) | *T* (^o^C) | *V*_pycnometer_ (cm^3^) | *ρ*_P25_ (g·cm^-3^) | Average *ρ*_P25_ (g·cm^-3^) | |
| --- | --- | --- | --- | --- | --- | --- | --- |
| 17.62498 | 21.05634 | 45.54405 | 20.01 |  | 3.681 |  | |
| 17.62499 | 21.05637 | 45.54385 | 20.00 |  | 3.680 |  | |
| 17.62496 | 21.05636 | 45.54403 | 20.00 | 25.4640 | 3.681 | 3.681 | |
| 17.62495 | 21.05635 | 45.54427 | 20.00 |  | 3.682 |  | |
| 17.62498 | 21.05634 | 45.54445 | 20.00 |  | 3.682 |  | |
| The standard uncertainty: *u*(*m*_3_)= 6.3×10^-5^ g, *u*(*m*_4_)= 6.2×10^-5^ g, *u*(*m*_5_)= ±1.6×10^-4^ g, *u*(*T*)= 0.02 K, The expanded uncertainty (*k* = 2, 95% confidence level): *U*_c_(*ρ*_P25_)= 8×10^-3^ g·cm^-3^. | | | | | | |  |

Table S10 Experimental data for the determination of P25 density using squalane as the solvent. *m*_3_: pycnometer weight, *m*_4_: weight of pycnometer plus P25, *m*_5_: weight of pycnometer plus sample plus a full bottle of squalane.

| *m*_3_ (g) | *m*_4_ (g) | *m*_5_ (g) | *T* (^o^C) | *V*_pycnometer_ (cm^3^) | *ρ*_P25_ (g·cm^-3^) | Average *ρ*_P25_ (g·cm^-3^) | |
| --- | --- | --- | --- | --- | --- | --- | --- |
| 16.45093 | 17.47738 | 25.33106 | 20.00 |  | 3.683 |  | |
| 16.45094 | 17.47736 | 25.33117 | 20.00 |  | 3.685 |  | |
| 16.45093 | 17.47737 | 25.33082 | 20.00 | 9.9926 | 3.679 | 3.681 | |
| 16.45092 | 17.47737 | 25.33065 | 20.00 |  | 3.676 |  | |
| 16.45094 | 17.47738 | 25.33091 | 20.00 |  | 3.680 |  | |
| The standard uncertainty: *u*(*m*_3_)= 6.3×10^-5^ g, *u*(*m*_4_)= 6.2×10^-5^ g, *u*(*m*_5_)= 1.4×10^-4^ g, *u*(*T*)= 0.02 K, The expanded uncertainty (*k* = 2, 95% confidence level): *U*_c_(*ρ*_P25_)= 7×10^-3^ g·cm^-3^. | | | | | | |  |

Table S11 The sample of P25-[Hmim][NTf_2_]-7.284%. *m*_6_: pycnometer weight, *m*_7_: weight of pycnometer plus sample, *m*_8_: weight of pycnometer plus sample plus a full bottle of squalane.

| *m*_6_ (g) | *m*_7_ (g) | *m*_8_ (g) | *T* (^o^C) | *V*_pycnometer_ (cm^3^) | *ρ*_P25-IL_  (g·cm^-3^) | Average *ρ*_P25-IL_  (g·cm^-3^) | |
| --- | --- | --- | --- | --- | --- | --- | --- |
| 11.29743 | 14.27291 | 21.88346 |  |  | 3.368 |  | |
| 11.29740 | 14.27290 | 21.88367 |  |  | 3.369 |  | |
| 11.29740 | 14.27290 | 21.88367 | 20.00 |  | 3.369 | 3.369 | |
| 11.29739 | 14.27290 | 21.88376 |  |  | 3.369 |  | |
| 11.29739 | 14.27289 | 21.88372 |  |  | 3.369 |  | |
| 11.29743 | 14.27291 | 21.82166 |  |  | 3.360 |  | |
| 11.29740 | 14.27290 | 21.82186 |  |  | 3.361 |  | |
| 11.29740 | 14.27290 | 21.82178 | 30.00 |  | 3.361 | 3.361 | |
| 11.29739 | 14.27290 | 21.82170 |  |  | 3.361 |  | |
| 11.29739 | 14.27289 | 21.82179 |  |  | 3.361 |  | |
| 11.29743 | 14.27291 | 21.76051 |  | 10.2967 | 3.356 |  | |
| 11.29740 | 14.27290 | 21.76081 |  |  | 3.357 |  | |
| 11.29740 | 14.27290 | 21.76054 | 40.00 |  | 3.356 | 3.356 | |
| 11.29739 | 14.27290 | 21.76027 |  |  | 3.355 |  | |
| 11.29739 | 14.27289 | 21.76031 |  |  | 3.355 |  | |
| 11.29743 | 14.27291 | 21.69961 |  |  | 3.352 |  | |
| 11.29740 | 14.27290 | 21.69940 |  |  | 3.351 |  | |
| 11.29740 | 14.27290 | 21.69972 | 50.00 |  | 3.352 | 3.351 | |
| 11.29739 | 14.27290 | 21.69949 |  |  | 3.351 |  | |
| 11.29739 | 14.27289 | 21.69945 |  |  | 3.351 |  | |
| The standard uncertainty: *u*(*m*_6_)= 6.3×10^-5^ g, *u*(*m*_7_)= 6.2×10^-5^ g, *u*(*m*_8_)= 1.2×10^-4^ g, *u*(*T*)= 0.02 K, The overall expanded uncertainty (*k* = 2, 95% confidence level) of the average density of P25-IL, *U*_c_(*ρ*_P25-IL_)= 9×10^-3^ g·cm^-3^. | | | | | | |  |

Table S12 The sample of P25-[Hmim][NTf_2_]-8.656%. m_6_: pycnometer weight, m_7_: weight of pycnometer plus sample, m_8_: weight of pycnometer plus sample plus a full bottle of squalane.

| *m*_6_ (g) | *m*_7_ (g) | *m*_8_ (g) | *T* (^o^C) | *V*_pycnometer_ (cm^3^) | *ρ*_P25-IL_  (g·cm^-3^) | Average *ρ*_P25-IL_  (g·cm^-3^) | |
| --- | --- | --- | --- | --- | --- | --- | --- |
| 10.93749 | 13.95769 | 21.65555 |  |  | 3.309 |  | |
| 10.93747 | 13.95770 | 21.65543 |  |  | 3.308 |  | |
| 10.93748 | 13.95770 | 21.65540 | 20.00 |  | 3.308 | 3.308 | |
| 10.93749 | 13.95768 | 21.65529 |  |  | 3.308 |  | |
| 10.93748 | 13.95769 | 21.65537 |  |  | 3.308 |  | |
| 10.93749 | 13.95769 | 21.59236 |  |  | 3.299 |  | |
| 10.93747 | 13.95770 | 21.59221 |  |  | 3.298 |  | |
| 10.93748 | 13.95770 | 21.59232 | 30.00 |  | 3.299 | 3.298 | |
| 10.93749 | 13.95768 | 21.59217 |  |  | 3.298 |  | |
| 10.93748 | 13.95769 | 21.59227 |  |  | 3.298 |  | |
| 10.93749 | 13.95769 | 21.53013 |  | 10.4339 | 3.293 |  | |
| 10.93747 | 13.95770 | 21.52991 |  |  | 3.292 |  | |
| 10.93748 | 13.95770 | 21.52985 | 40.00 |  | 3.291 | 3.292 | |
| 10.93749 | 13.95768 | 21.52999 |  |  | 3.292 |  | |
| 10.93748 | 13.95769 | 21.53007 |  |  | 3.292 |  | |
| 10.93749 | 13.95769 | 21.46781 |  |  | 3.285 |  | |
| 10.93747 | 13.95770 | 21.46774 |  |  | 3.285 |  | |
| 10.93748 | 13.95770 | 21.46792 | 50.00 |  | 3.286 | 3.285 | |
| 10.93749 | 13.95768 | 21.46778 |  |  | 3.285 |  | |
| 10.93748 | 13.95769 | 21.46789 |  |  | 3.286 |  | |
| The standard uncertainty: *u*(*m*_6_)= 6.2×10^-5^ g, *u*(*m*_7_)= 6.2×10^-5^ g, *u*(*m*_8_)= 1.3×10^-4^ g, *u*(*T*)= 0.02 K, The expanded uncertainty (*k* = 2, 95% confidence level) of the average density of P25-IL, *U*_c_(*ρ*_P25-IL_)= 9×10^-3^ g·cm^-3^. | | | | | | |  |

Table S13 The sample of P25-[Hmim][NTf_2_]-11.962%. *m*_6_: pycnometer weight, *m*_7_: weight of pycnometer plus sample, *m*_8_: weight of pycnometer plus sample plus a full bottle of squalane.

| *m*_6_ (g) | *m*_7_ (g) | *m*_8_ (g) | *T* (^o^C) | *V*_pycnometer_ (cm^3^) | *ρ*_P25-IL_  (g·cm^-3^) | Average *ρ*_P25-IL_  (g·cm^-3^) | |
| --- | --- | --- | --- | --- | --- | --- | --- |
| 10.59115 | 13.72469 | 21.47054 |  |  | 3.147 |  | |
| 10.59116 | 13.72470 | 21.47067 |  |  | 3.147 |  | |
| 10.59114 | 13.72470 | 21.47081 | 20.00 |  | 3.148 | 3.147 | |
| 10.59116 | 13.72470 | 21.47060 |  |  | 3.147 |  | |
| 10.59115 | 13.72469 | 21.47072 |  |  | 3.148 |  | |
| 10.59115 | 13.72469 | 21.40753 |  |  | 3.140 |  | |
| 10.59116 | 13.72470 | 21.40766 |  |  | 3.141 |  | |
| 10.59114 | 13.72470 | 21.40766 | 30.00 |  | 3.141 | 3.141 | |
| 10.59116 | 13.72470 | 21.40760 |  |  | 3.141 |  | |
| 10.59115 | 13.72469 | 21.40762 |  |  | 3.141 |  | |
| 10.59115 | 13.72469 | 21.34427 |  | 10.5762 | 3.132 |  | |
| 10.59116 | 13.72470 | 21.34469 |  |  | 3.134 |  | |
| 10.59114 | 13.72470 | 21.34433 | 40.00 |  | 3.133 | 3.133 | |
| 10.59116 | 13.72470 | 21.34440 |  |  | 3.133 |  | |
| 10.59115 | 13.72469 | 21.34425 |  |  | 3.132 |  | |
| 10.59115 | 13.72469 | 21.28215 |  |  | 3.128 |  | |
| 10.59116 | 13.72470 | 21.28115 |  |  | 3.127 |  | |
| 10.59114 | 13.72470 | 21.28203 | 50.00 |  | 3.128 | 3.128 | |
| 10.59116 | 13.72470 | 21.28210 |  |  | 3.128 |  | |
| 10.59115 | 13.72469 | 21.28197 |  |  | 3.128 |  | |
| The standard uncertainty: *u*(*m*_6_)= 6.2×10^-5^ g, *u*(*m*_7_)= 6.2×10^-5^ g, *u*(*m*_8_)= 1.2×10^-4^ g, *u*(*T*)= 0.02 K, The expanded uncertainty (*k* = 2, 95% confidence level) of the average density of P25-IL, *U*_c_(*ρ*_P25-IL_)= 9×10^-3^ g·cm^-3^. | | | | | | |  |

Table S14 The sample of P25-[Hmim][NTf_2_]-15.195%. *m*_6_: pycnometer weight, *m*_7_: weight of pycnometer plus sample, *m*_8_: weight of pycnometer plus sample plus a full bottle of squalane.

| *m*_6_ (g) | *m*_7_ (g) | *m*_8_ (g) | *T* (^o^C) | *V*_pycnometer_ (cm^3^) | *ρ*_P25-IL_  (g·cm^-3^) | Average *ρ*_P25-IL_  (g·cm^-3^) | |
| --- | --- | --- | --- | --- | --- | --- | --- |
| 14.87821 | 18.13127 | 24.22231 |  |  | 2.979 |  | |
| 14.87823 | 18.13126 | 24.22220 |  |  | 2.979 |  | |
| 14.87825 | 18.13128 | 24.22223 | 20.00 |  | 2.979 | 2.979 | |
| 14.87824 | 18.13127 | 24.22228 |  |  | 2.979 |  | |
| 14.87822 | 18.13127 | 24.22247 |  |  | 2.979 |  | |
| 14.87821 | 18.13127 | 24.17309 |  |  | 2.976 |  | |
| 14.87823 | 18.13126 | 24.17272 |  |  | 2.974 |  | |
| 14.87825 | 18.13128 | 24.17315 | 30.00 |  | 2.976 | 2.976 | |
| 14.87824 | 18.13127 | 24.17299 |  |  | 2.975 |  | |
| 14.87822 | 18.13127 | 24.17346 |  |  | 2.977 |  | |
| 14.87821 | 18.13127 | 24.12486 |  | 8.6258 | 2.975 |  | |
| 14.87823 | 18.13126 | 24.12289 |  |  | 2.969 |  | |
| 14.87825 | 18.13128 | 24.12296 | 40.00 |  | 2.969 | 2.970 | |
| 14.87824 | 18.13127 | 24.12287 |  |  | 2.968 |  | |
| 14.87822 | 18.13127 | 24.12280 |  |  | 2.968 |  | |
| 14.87821 | 18.13127 | 24.07345 |  |  | 2.964 |  | |
| 14.87823 | 18.13126 | 24.07376 |  |  | 2.965 |  | |
| 14.87825 | 18.13128 | 24.07321 | 50.00 |  | 2.963 | 2.964 | |
| 14.87824 | 18.13127 | 24.07348 |  |  | 2.964 |  | |
| 14.87822 | 18.13127 | 24.07361 |  |  | 2.964 |  | |
| The standard uncertainty: *u*(*m*_6_)= 6.2×10^-5^ g, u(*m*_7_)= 6.2×10^-5^ g, *u*(*m*_8_)= 1.7×10^-4^ g, *u*(*T*)= 0.02 K, The expanded uncertainty (*k* = 2, 95% confidence level) of the average density of P25-IL, *U*_c_(*ρ*_P25-IL_)= 9×10^-3^ g·cm^-3^. | | | | | | |  |

Table S15 The sample of P25-[Hmim][NTf_2_]-30.195%. *m*_6_: pycnometer weight, *m*_7_: weight of pycnometer plus sample, *m*_8_: weight of pycnometer plus sample plus a full bottle of squalane.

| *m*_6_ (g) | *m*_7_ (g) | *m*_8_ (g) | *T* (^o^C) | *V*_pycnometer_ (cm^3^) | *ρ*_P25-IL_  (g·cm^-3^) | Average *ρ*_P25-IL_  (g·cm^-3^) | |
| --- | --- | --- | --- | --- | --- | --- | --- |
| 11.29754 | 15.24965 | 22.28100 |  |  | 2.470 |  | |
| 11.29753 | 15.24965 | 22.28110 |  |  | 2.470 |  | |
| 11.29754 | 15.24963 | 22.28129 | 20.00 |  | 2.471 | 2.471 | |
| 11.29754 | 15.24965 | 22.28123 |  |  | 2.471 |  | |
| 11.29752 | 15.24962 | 22.28117 |  |  | 2.471 |  | |
| 11.29754 | 15.24965 | 22.22028 |  |  | 2.461 |  | |
| 11.29753 | 15.24965 | 22.22041 |  |  | 2.461 |  | |
| 11.29754 | 15.24963 | 22.22012 | 30.00 |  | 2.460 | 2.461 | |
| 11.29754 | 15.24965 | 22.22018 |  |  | 2.460 |  | |
| 11.29752 | 15.24962 | 22.22025 |  |  | 2.461 |  | |
| 11.29754 | 15.24965 | 22.15917 |  | 10.2967 | 2.450 |  | |
| 11.29753 | 15.24965 | 22.15944 |  |  | 2.450 |  | |
| 11.29754 | 15.24963 | 22.15920 | 40.00 |  | 2.450 | 2.450 | |
| 11.29754 | 15.24965 | 22.15932 |  |  | 2.450 |  | |
| 11.29752 | 15.24962 | 22.15930 |  |  | 2.450 |  | |
| 11.29754 | 15.24965 | 22.09904 |  |  | 2.441 |  | |
| 11.29753 | 15.24965 | 22.09881 |  |  | 2.440 |  | |
| 11.29754 | 15.24963 | 22.09888 | 50.00 |  | 2.441 | 2.441 | |
| 11.29754 | 15.24965 | 22.09898 |  |  | 2.441 |  | |
| 11.29752 | 15.24962 | 22.09901 |  |  | 2.441 |  | |
| The standard uncertainty: *u*(*m*_6_)= 6.2×10^-5^ g, *u*(*m*_7_)= 6.2×10^-5^ g, *u*(*m*_8_)= 1.3×10^-4^ g, *u*(*T*)= 0.02 K, The expanded uncertainty (*k* = 2, 95% confidence level) of the average density of P25-IL, *U*_c_(*ρ*_P25-IL_)= 9×10^-3^ g·cm^-3^. | | | | | | |  |

In the density measurements for the heterogeneous system with a pycnometer, the error sources mainly include the uncertainty of temperature *u*(*T*), reagent purity *u*(*P*), measurement uncertainty of instrument, and the uncertainty of mass *u*(*m*). The uncertainty to the final objective function *u*(*ρ*) can be analyzed through the error propagation analysis(Javed et al, 2020; Lorefice et al, 2014). The uncertainty of temperature comes from a thermometer and a constant temperature water bath, which is *u*(*T*)= 0.02 K. The mass is determined by an electronic analytical balance, which mainly includes the resolution of the balance, the maximum allowable error, the eccentric load measurement of the balance, and the repeatability. The repeatability is expressed by the standard deviation. The total expanded uncertainty of weight is *u*(*m*)= 6.2×10^-5^ g. The error analysis of the measured data at 293.15 K was used as an example, and the analysis process is the same for those measured at other temperatures.

Table S16 Analysis of the sources of error in electronic balances.

| Source of uncertainty | Standard uncertainty | Probability distribution | | Uncertainty contribution (g) | |
| --- | --- | --- | --- | --- | --- |
| Repeatability (type A) | *u*_rep_(*m*) | normal | | 2.0×10^-5^ | |
| Readability (type B) | *u*_read_(*m*) | square | | 2.9×10^-6^ | |
| Eccentricity test (type B) | *u*_ecc_(*m*) | square | | 9.6×10^-6^ | |
| Linearity test (type B) | *u*_line_(*m*) | square | | 5.8×10^-5^ | |
| Combined standard uncertainty *u*(*m*) | | | 6.2×10^-5^ | |  |

Error analysis of pycnometers

$V_{i-pycnometer}=\frac{m_{2}-m_{1}}{\rho_{H_{2}O}}$ (S1)

$$u(V_{i-pycnometer})=\sqrt{(\frac{\partial V_{i-pycnometer}}{\partial m_{1}}\times u(m_{1}))^{2}+(\frac{\partial V_{i-pycnometer}}{\partial m_{2}}\times u(m_{2}))^{2}+(\frac{\partial V_{i-pycnometer}}{\partial\rho_{H_{2}O}}\times u(\rho_{H_{2}O}))^{2}}$$

(i = 1, 2, 3, 4, 5, 6) (S2)

Table S17 Sources of error and error analysis of pycnometers.

| Pycnometer | *u* (*m*_1_) (g) | *u* (*m*_2_) (g) | *u* (*ρ*_H2O_) (g·cm^-3^) | *U*_c_ (*V*_i-pycnometer_) (cm^3^) |
| --- | --- | --- | --- | --- |
| 1 | 6.3×10^-5^ | 1.3×10^-4^ | 5×10^−6^ | 4×10^−4^ |
| 2 | 6.3×10^-5^ | 1.3×10^-5^ | 5×10^-6^ | 4×10^−4^ |
| 3 | 6.3×10^-5^ | 1.5×10^-4^ | 5×10^−6^ | 3×10^−4^ |
| 4 | 6.2×10^-5^ | 1.2×10^-4^ | 5×10^−6^ | 3×10^−4^ |
| 5 | 6.2×10^-5^ | 1.2×10^-4^ | 5×10^−6^ | 3×10^−4^ |
| 6 | 6.3×10^-5^ | 2.2×10^-4^ | 5×10^−6^ | 5×10^−4^ |

Error analysis of the density of P25

$\rho_{P25}=\frac{(m_{4}-m_{3})\times\rho_{solvent}}{(m_{2}-m_{1})\times(m_{5}-m_{4})}$ (S3)

(S4)

Table S18 The sources of measurement error and error analysis of the density of P25 using water or squalane as solvent.

| *u* (*m*_3_) (g) | *u* (*m*_4_) (g) | *u* (*m*_5_) (g) | *u*_p_ (P25) (g·g^-1^) | *U*_c_(ρ_TiO2_)$\text{U}\text{c }\text{(}\text{ρ}_{\text{Ti}_{\text{2}}\text{O}}\text{) }$ (g·cm^-3^) |
| --- | --- | --- | --- | --- |
| 6.3×10^-5^ | 6.2×10^-5^ | 1.6×10^-4^ | 0.0014 | 0.008 |
| 6.3×10^-5^ | 6.2×10^-5^ | 1.4×10^-4^ | 0.0014 | 0.007 |

Error analysis of the density of P25-IL

$\rho_{P25-IL}=\frac{(m_{7}-m_{6})}{\frac{(m_{2}-m_{1})}{\rho_{H_{2}O}}-\frac{(m_{8}-m_{7})}{\rho_{squalane}}}$ (S5)

(S6)

Table S19 The sources of measurement error and error analysis of the density of P25-IL.

| P25-IL (wt%) | *u* (*m*_6_)  (g) | *u* (*m*_7_)  (g) | *u* (*m*_8_)  (g) | *u* (*ρ*_squalane_)  (g·cm^-3^) | *u*_p_(squalane)  (g·g^-1^) | *u*_p_(P25)  (g·g^-1^) | *u*_p_ (IL)  (g·g^-1^) | *U*_c_(ρ_TiO2-IL_)  (g·cm^-3^) |
| --- | --- | --- | --- | --- | --- | --- | --- | --- |
| 7.284 | 6.3×10^-5^ | 6.2×10^-5^ | 1.3×10^-4^ | 6.4×10^-6^ | 0.0029 | 0.0014 | 0.0029 | 0.009 |
| 8.656 | 6.2×10^-5^ | 6.2×10^-5^ | 1.3×10^-4^ | 6.4×10^-6^ | 0.0029 | 0.0014 | 0.0029 | 0.009 |
| 11.962 | 6.2×10^-5^ | 6.2×10^-5^ | 1.3×10^-4^ | 6.4×10^-6^ | 0.0029 | 0.0014 | 0.0029 | 0.009 |
| 15.195 | 6.3×10^-5^ | 6.2×10^-5^ | 1.2×10^-4^ | 6.4×10^-6^ | 0.0029 | 0.0014 | 0.0029 | 0.009 |
| 30.195 | 6.3×10^-5^ | 6.2×10^-5^ | 1.3×10^-4^ | 6.4×10^-6^ | 0.0029 | 0.0014 | 0.0029 | 0.009 |

Error analysis of the volume of P25

$V_{P25}=\frac{(m_{2}-m_{1}+m_{5}-m_{4})\times a\times(m_{7}-m_{6})}{(m_{4}-m_{3})\times\rho_{H_{2}O}}$ (a is the mass fraction of P25) (S7)

(S8)

Error analysis of the volume of squalane

*$V_{squalane}=\frac{m_{squalane}}{\rho_{squalane}}=\frac{m_{8}-m_{7}}{\rho_{squalane}}$* (S9)

*$u(V_{squalane})=\sqrt{(\frac{\partial V_{squalane}}{\partial m_{7}}\times u(m_{7}))^{2}+(\frac{\partial V_{squalane}}{\partial m_{8}}\times u(m_{8}))^{2}+(\frac{\partial V_{squalane}}{\partial\rho_{squalane}}\times u(\rho_{squalane}))^{2}}$* (S10)

Table S20 Error analysis of the volume of squalane.

| P25-IL (wt%) | *U*_c_ (*V*_squalane_) (cm^3^) |
| --- | --- |
| 7.284 | 0.0004 |
| 8.656 | 0.0004 |
| 11.962 | 0.0004 |
| 15.195 | 0.0004 |
| 30.195 | 0.0004 |

The sources of measurement error and error analysis of the mole of IL, volume of interface IL and molar volume of interface IL.

$V_{interface-IL}=\frac{m_{2}-m_{1}}{\rho_{H_{2}O}}-\frac{(m_{7}-m_{6})\times a\times(m_{2}-m_{1}+m_{4}-m_{5})}{(m_{4}-m_{3})\times\rho_{H_{2}O}}-\frac{m_{8}-m_{7}}{\rho_{squalane}}$ (S11)

 (S12)

$n_{IL}=\frac{(m_{8}-m_{7})\times(1-a)}{M_{IL}}$ (S13)

$u(n_{IL})=\frac{(1-a)}{M_{IL}}\sqrt{(u(m_{7}))^{2}+(u(m_{8}))^{2}}$ (S14)

Molar volume of ionic liquid immobilized on the surface of P25: *V*_m-interface-IL_

$V_{m-IL-interface}=\frac{V_{IL-interface}}{n_{IL}}$ (S15)

$u(V_{m-interface-IL})=V_{m-interface-IL}\times\sqrt{(\frac{u(V_{interface-IL})}{V_{interface-IL}})^{2}+(\frac{u(n_{IL})}{n_{IL}})^{2}}$ (S16)

$U_{c}(V_{m-IL-interface})=k\times u(V_{m-IL-interface})$(*k* = 2, 95% confidence level) (S17)

Table S21 The error analysis of the mole of IL, volume of interface IL and molar volume of interface IL.

| P25-IL (wt%) | 10^6^×u (*n*_IL_)  (mol) | *u* (*V*_interface-IL_)  (cm^3^) | *V*_m-interface-IL_  (cm^3^·mol^-1^) | *U*_c_ (*V*_m-interface-IL_)  (cm^3^·mol^-1^) |
| --- | --- | --- | --- | --- |
| 7.284 | 0.02 | 0.006 | 276 | 18 |
| 8.656 | 0.03 | 0.006 | 279 | 15 |
| 11.962 | 0.04 | 0.006 | 293 | 10 |
| 15.195 | 0.05 | 0.006 | 310 | 8 |
| 30.195 | 0.10 | 0.006 | 318 | 3 |

The expanded uncertainty of standard molar volume of interface ionic liquids

$V_{m-interface-IL}^{\infty}=\lim_{n_{IL}\to0} \frac{V_{m-interface-IL}}{n_{IL}}$ (S18)

$u(V_{m-interface-IL}^{\infty})=V_{m-interface-IL}^{\infty}\times\sqrt{(\frac{u(V_{m-interface-IL}^{\infty})}{V_{m-interface-IL}})^{2}+(\frac{u(n_{IL})}{n_{IL}})^{2}}$ (S19)

$U_{c}(V_{m-IL-interface}^{\infty})=k\times u(V_{m-IL-interface}^{\infty})$(*k* = 2, 95% confidence level) (S20)

# Supplementary Figures





# Supplementary Figure 1. ^1^H NMR(DMSO) spectra for [Hmim][NTf_2_].





**Supplementary Figure 2**. XRD patterns of P25 and P25-IL samples.

|  |  |
| --- | --- |
|  |  |

**Supplementary Figure 3.** N_2_ adsorption−desorption isotherms as well as pore volumes and pore size distributions of P25 and P25-IL.





**Supplementary Figure 4.** FT-IR spectra for upper solutions of the ternary (P25-IL-squalane) systems and squalane.

|  |  |
| --- | --- |

**Supplementary Figure 5.** UV-vis absorption spectra of the mixed solution of ILs and squalane. (a) Determination of the maximum absorbance of the mixed solution; (b) UV-vis spectral absorbance for the solutions of ILs and squalane with different mass ratios.

**References**

Javed, M. A., Rüther, M., Baumhögger, E.,Vrabec, J. (2020) Density and Thermodynamic Speed of Sound of Liquid Vinyl Chloride. *J. Chem. Eng. Data.*, 65, 2495-2504.doi:10.1021/acs.jced.9b01133

Lorefice, S., Romeo, R., Santiano, M.,Capelli, A. (2014) Original pycnometers for volatile liquid density over wide ranges of temperature and pressure: practical example. *Metrologia*, 51, 154-160.doi:10.1088/0026-1394/51/3/154
